# Plenary Speaker Abstracts

**DOI:** 10.1080/24740527.2018.1476315

**Published:** 2018-05-21

We were taught, and we teach our children, that there are five senses. This unusual perspective on sensation and experience extends into adulthood and into professional education. The psychology of perception, for example, focusses almost exclusively on vision. I present here an invitation to explore the ten neglected senses, of balance, motion, pressure, itch, pain, fatigue, breathing, temperature, appetite, and expulsion (the experience of matter leaving the body). Clinically, the experiences of the body, the physical senses are what are most often at stake, they form the content of the patient reported outcomes argued to be central to chronic healthcare.

A functional and phenomenological account of embodied (interoceptive and proprioceptive) experience is presented, focusing on what the function of a specific experience is, what consequences it leads to, and how it feels. Bringing a formal psychological frame to this experience, using scientific method, can bring into focus opportunities for clinical intervention and improvements in patient experience (Eccleston, 2016).

Using the three examples of pain, itch, and respiration, embodied perception is explored. First, non-clinical limit (extreme) experience are explored, second, what we know from experimental research is reviewed, and finally, the clinical consequences of each sense is developed and exemplified. In particular the learnings for the next generation of self-management, psychological and rehabilitative treatments will be outlined (Eccleston and Crombez, 2017).
